# Supplementary material for: Evaluating the Effectiveness of Visuospatial Memory Stimulation Using Virtual Reality in Head and Neck Cancer Patients—Pilot Study
Source: Cancers (Basel). 2023 Mar 7;15(6):1639. doi: 10.3390/cancers15061639 (PMC10046573; doi:10.3390/cancers15061639)
Supplement: Supplementary file 1 [file cancers-15-01639-s001.zip › cancers-2211116-supplementary.pdf]

Supplementary Materials

Schedule of interventions.

Session numer	**Application title**	**Characteristics**
1	The Blue	Adventure, Casual, Exploration
2	The Coast	Casual, Music, Music Video
3	The Grand Canyon Day	Casual, Exploration, Travel
4	The Art. Plunge	Art/Creativity, Relaxation/Meditation
5	Nature Treks 1	Exploration, Relaxation/Meditation, Simulation
6	The Night Cafe	Art/Creativity, Casual, Educational, Exploration
7	The Grand Canyon Night	Casual, Exploration, Travel
8	Transition	Music Video, Relaxation/Meditation
9	Perfect Mountain	Casual, Exploration, Relaxation/Meditation, Simulation
10	Nature Treks 2	Exploration, Relaxation/Meditation, Simulation
11	Henry	Animation/Family/Entertainment
12	Perfect Lake	Casual, Exploration, Relaxation/Meditation, Simulation
13	Luna 1	Casual, Exploration, Music, Narrative, Puzzle
14	Oculus First Contact	Casual, Exploration
15	Nature Treck 3	Exploration, Relaxation/Meditation, Simulation
16	Paint VR	Art/Creativity, Casual
17	Perfect Forest	Casual, Exploration, Relaxation/Meditation, Simulation
18	Nature Treck 4	Exploration, Relaxation/Meditation, Simulation
19	Luna 2	Casual, Exploration, Music, Narrative, Puzzle
20	The Crow	Movie, Narrative
21	Drops: Rhythm Garden	Music, Relaxation/Meditation, Utility
22	Nature Treck 5	Exploration, Relaxation/Meditation, Simulation
23	Luna 3	Casual, Exploration, Music, Narrative, Puzzle
